# Traumatic events and posttraumatic stress symptoms in a treatment-seeking sample of Ukrainian children during the war

**DOI:** 10.1186/s13034-024-00715-1

**Published:** 2024-02-09

**Authors:** Elisa Pfeiffer, Maike Garbade, Cedric Sachser

**Affiliations:** Department of Child and Adolescent Psychiatry/ Psychotherapy, Ulm University, Steinhoevelstr. 2, 89075 Ulm, Germany

**Keywords:** Trauma, Children, PTSD, Ukraine, War

## Abstract

**Background:**

The Russian invasion of Ukraine resulted in a dramatic increase of children and adolescents being confronted with war and other traumatic experiences, which could result in an increase of trauma-related mental health disorders such as posttraumatic stress disorder (PTSD) in an entire generation. This study aims at reporting the prevalence of traumatic events, PTSD, and Complex PTSD (CPTSD) in children and adolescents seeking for mental health treatment since the Russian invasion. Additionally, the consistency of child and caregiver reported trauma, PTSD and CPTSD will be examined.

**Methods:**

This study is part of the “TF-CBT Ukraine” project in which Ukrainian therapists were trained in assessing their patients via the “Child and Adolescent Trauma Screen” (CATS-2) before initiating trauma-focused treatment, if indicated. Altogether *N* = 200 Ukrainian children and adolescents (*M*_age_ = 12.01, range 4–21; 62.0% female) were included in the study between October 2022 and August 2023. Data were analysed descriptively, via t-tests and bivariate correlations.

**Results:**

The children and adolescents reported on average four different traumatic events, most frequently war (*n* = 123; 68.7%), bullying threats (*n* = 71; 39.7%) and domestic violence (*n* = 68; 38.0%). Almost 70% (*n* = 123) of the participants fulfilled the DSM-5 PTSD criteria, 31% (*n* = 56) fulfilled the ICD-11 PTSD criteria and 21% (*n* = 38) the ICD-11 CPTSD criteria. Rates of PTSD were even higher in preschool children (95%). The comparisons of self-and caregiver reports on traumatic events and PTSD/CPTSD severity scores indicated moderate to high correlations between the patients and their caregivers (*r* = 0.710–0.767).

**Conclusions:**

This study shows that Ukrainian children and adolescents starting treatment report a high number of traumatic events and trauma-related symptoms, which could have a long-lasting negative impact on their social-emotional development and quality of life. The implementation of evidence-based trauma-focused interventions for these children is therefore crucial.

**Supplementary Information:**

The online version contains supplementary material available at 10.1186/s13034-024-00715-1.

## Introduction

Since the large-scale Russian invasion into Ukraine started in February 2022, countless civilians have been killed, and millions of children and families had to either live in a war-torn country, constantly being under attack, or had to flee the country which means leaving behind their entire life. The burden of mental health disorders is known to be high in conflict-affected populations [1], but studies on prevalence rates of mental health disorders in war-affected children and adolescents are comparably rare and rates vary widely. For example, prevalence rates of posttraumatic stress disorder (PTSD) in adolescents in conflict/ war-torn regions such as Syria [2], Israel [3] or Palestine [4] vary between 7.6 and 53%. These wide variations could be explained by the very different contexts, methods employed and target populations. Across many studies, though, several factors have been found to influence the development of trauma-related disorders in children and adolescents, most common are sex, age, number and intensity of traumatic events, social support or socioeconomic status [5].

Especially since rates of child abuse and neglect, and consequently reduced general and mental health in Ukrainian children had been reported even before the war [6], we now need a better understanding of the trauma history and PTSD symptoms in this population, in order to implement and evaluate needs-oriented interventions for these children. A first study by Karatzias et al. [7] investigated adult and parent-reported mental health data of children in Ukraine via a nationwide sample of 2004 adult parents from the general population. They found that 25% of the parents met PTSD criteria and 14.6% met criteria for Complex Posttraumatic stress disorder (CPTSD). When asked about their child’s PTSD symptoms (assessed via caregiver-report) 18.5% of pre-schoolers and 14.2% of school-age children fulfilled PTSD criteria based on the “Child and Adolescent Trauma Screen” (CATS) [8] caregiver screening measure [9]. Unfortunately, there was no self-reported trauma history or PTSD symptom screening of children and adolescents in this well conducted survey, as the main target group were adults. However, as internalizing symptoms are more difficult for parents to observe, it is important in the screening and diagnosis of children and adolescents that both perspectives are considered. However, another study by Osokina et al. 2023 [10] report the war-related trauma history and PTSD in a cross-sectional study which included 2766 adolescents aged 11–17 years in the war-torn region Donetsk and in Kirovograd, a region that hadn’t been affected by war during study conduction (2016–2017). They found high rates of war-related trauma such as witnessing armed attacks (60.2%), being forced to leave their hometowns (27.9%) or seeking safer places/hiding in basements/air raid shelters (26.7%) in adolescents in the war-torn region. Adolescents in the war-torn regions reported higher rates of PTSD (5.3% vs. 1.2%), depression (7.5% vs. 2.9%) and anxiety (4.4% vs. 1.5%) compared with adolescents in the non-war region. Direct and indirect exposure to war was associated with an elevated risk for mental health problems, which is in line with other studies investigating adolescents living in war-related atrocities [11]. The high rates of PTSD in Ukrainian civilians after the war started in 2014 was also confirmed in a study conducted 36 months after the conflict began, showing that Ukrainian adults who had been internally displaced due to the Russian invasion in 2014 showed even higher rates of PTSD, compared with Ukrainians who were also affected by the war but who were not internally displaced [12]. Since the last two studies were conducted, the entire country has been affected by the war and children and adolescents all over Ukraine were exposed to high levels of traumatic events, which may result in higher prevalence of psychological distress and development of trauma-related disorders such as PTSD.

To our knowledge, there is no study investigating Ukrainian children and adolescents’ self-and caregiver-reported trauma history and their PTSD/ CPTSD prevalence rates since 2022. Hence, this study aims at filling this gap in the literature by reporting cross-sectional data of trauma and PTSD/CPTSD in Ukrainian children and adolescents, assessed between October 2022 and August 2023 by their therapists. Based on the current literature, we hypothesize that the children and adolescents in this study report high rates of traumatic events and high prevalence rates of PTSD and CPTSD as they are living under current threat. Additionally, self- and caregiver reports of trauma history and PTSS will be compared to investigate the parents’ awareness of the child’s symptoms and trauma history.

## Methods

This study is part of the “TF-CBT Ukraine” project [13] which aims to implement and evaluate the evidence-based trauma-focused treatment “Trauma-focused Cognitive Behavioral Therapy” (TF-CBT) [14] in Ukraine during the war. This project builds on Ukrainian as well as international collaborations with the TF-CBT treatment developers, certified international TF-CBT trainers, the National Psychological Association of Ukraine, the National Child Traumatic Stress Network (USA), the Ministry of Health of Ukraine and Ministry of Education and Science of Ukraine, and the “Mental health for Ukraine Project” implemented by GFA Consulting Group GmbH. This project received ethical approval by the Ulm University (Number: Cl/Sta) in Germany and the Zhytomyr Ivan Franko State University (Number: 9–08072022) in Ukraine. The project started in March 2022 and is anticipated to end in May 2024.

The Ukrainian therapists in the TF-CBT Ukraine project received a training on how to implement, analyze and interpret the CATS-2 measure. When treating patients with TF-CBT, the therapists were asked to assess each child/ adolescent and their caregiver before and after the treatment with the CATS-2 measure. The data was then entered anonymously into an online survey by the therapist.

### Participants

Participants were recruited by *n* = 67 therapists in their current work settings. They invited the children/ adolescents and caregivers to the assessment and TF-CBT treatment. There were no distinct criteria for children and adolescents who could be treated by the therapists. However, therapists learnt during their basic trainings in the program that TF-CBT patients should be between 4–21 years old, have experienced a traumatic event and report at least moderate PTSD symptoms in order to benefit from the treatment.

### Measures

*Child and Adolescent Trauma Screen* Second Version (CATS-2) measures potentially traumatic events (PTEs) and posttraumatic stress symptoms (PTSS). For children and adolescents from 7 to 21 years the second version of the CATS (CATS-2) [15] was used, which allowed the analysis of PTSS according to DSM-5 and ICD-11 criteria. First, the experience of PTEs is assessed using a 15-item structured PTE checklist, including diverse events, such as the experience or witness of violence at home, sexual abuse or war. Participants can indicate whether they had experienced the event by indicating either “yes” or “no”. Subsequently, PTSS in the last four weeks are assessed using 25 items rated on a 4-point Likert scale (0 = “Never”, 1 =  “Sometimes”, 2 =  “Often”, 3 =  “Almost Always”). Psychosocial functioning was measured via five items (“yes”/ “no”) which ask whether the previously reported PTSS interfere with five key areas of functioning (getting along with others, school/work, hobbies, family relationships, and general happiness). The reliability in the current study was fair to good (α_DSM-5 PTSD_ = 0.89; α_ICD-11 PTSD_ = 0.69; α_ICD-11 CPTSD_ = 0.78). In addition to the self-report measure for children and adolescents, the caregivers were asked to fill out a parallel caregiver version (α_DSM-5 PTSD_ = 0.92; α_ICD-11 PTSD_ = 0.79; α_ICD-11 CPTSD_ = 0.84).

For preschool children aged between 3 and 6 years, the caregiver version of the CATS [8] was used, as there is no adapted version of the CATS-2 for preschool children. The caregiver version of the CATS for preschool children includes the same 15-item PTEs checklist as the CATS-2. Subsequently, PTSS in the last two weeks are measured by 16 items rated on a 4-point Likert scale (0 = “Never”, 1 = “Sometimes”, 2 = “Often”, 3 = “Almost always”) (α_DSM-5 PTSD_ = 0.67). Psychosocial functioning was assessed using the same five yes/no items as in the CATS-2.

*Socio-demographic information* such as age, gender and current location was assessed via an unstandardized questionnaire.

### Statistical analyses

Data analyses were conducted using IBM SPSS Statistics for Windows, Version 28.0. Descriptive statistics (means, standard deviation, ranges, and frequencies) were computed to profile the sociodemographics. To explore the PTSD/CPTSD-severity scores, the items of the CATS-2 were summed up as recommended by Sachser et al. [15]. The DSM-5 severity score was calculated by summing up the items 1–20 (range 0–60), including only the highest score of items 9, 10 and 15. The ICD-11 PTSD severity score (range 0–18) was calculated by summing up the items 2, 3, 6, 7, 17 and 18. The ICD-11 CPTSD score (range 0–36) is the sum of the ICD-11 PTSD severity score plus the sum of the ICD-11 Disturbances in self-organization severity score (9b, 9d, 10a, 13, 14, 15a). To examine, how many participants fulfill the clinical criteria for PTSD and CPTSD, the categorical item-mapping approach of the CATS-2 was followed [15]. This approach follows the diagnostic algorithms of the DSM-5 and ICD-11 with a symptom being rated as present for values of 2 = “Often” or 3 = “Almost Always”.

To explore the PTSD-severity scores in preschool children, the items of the CATS were summed up (range 0–48) and the categorical item-mapping approach was used to examine how many preschoolers fulfilled the clinical criteria for PTSD according to the DSM-5 criteria [8].

In order to examine the match between self-report and caregiver-report, bivariate correlations (Pearson correlation coefficients (r)) between self- and caregiver reports of the CATS-2 DSM-5 PTSD-sum score and the CATS-2 ICD-11 PTSD/CPTSD-sum scores and Φ-correlations between the single events of the CATS-2 event scale were calculated.

## Results

### Sample description

The study sample consisted of *N* = 200 children, adolescents and young adults (*n* = 76 (38.0%) male; *M*_age_ = 12.01; *SD* = 4.05; range 4–21; *n* = 9 (4.5%) were older than 17 years old)), including *n* = 21 (10.5%) preschool children (between 3 and 6 years old). The majority of the participants (*n* = 115, 62.5%) were still located in Ukraine. Demographic information of the entire study sample, also separated by age (preschoolers aged 3–6 vs. participants aged 7+), is presented in Table 1.

**Table 1:** Descriptive characteristics of the study sample

	Total sample (*N* = 200)	Age 7 + (*n* = 179)	Age 3–6 (*n* = 21)
	*M* (*SD*)	*M* (*SD*)	*M* (*SD*)
Age	12.01 (4.05) Range 4–21	12.79 (3.54) Range 7–21	5.38 (0.81) Range 4–6
Gender (female) (*n, %)*	124 (62.0)	110 (61.5)	14 (66.7)
Current location (*n, %)*			
Inside Ukraine	126 (63.0)	111 (62.0)	15 (71.4)
Outside Ukraine	62 (31.0)	56 (31.3)	6 (28.6)
Unknown	12 (6.0)	12 (6.7)	–
Present Caregiver^a^ (*n, %)*			
Mother	156 (78.0)	136 (76.0)	20 (95.2)
Father	32 (16.0)	31 (17.3)	1 (4.8)
Grandparent	17 (8.5)	16 (8.9)	1 (4.8)
Professional (e.g. child welfare staff)	7 (3.5)	6 (3.4)	1 (4.8)
Other	13 (6.0)	11 (6.1)	1 (4.8)

^***a***^Multiple answers were possible

### Trauma

The participants aged 7 years or older reported on average 4.23 different PTEs (*SD* = 2.54, range 0–12). The most frequently reported PTEs were “war” (*n* = 112, 68.7%), “bullying” (*n* = 71, 39.7%), and “witnessing domestic violence” (*n* = 68, 38.9%). The least frequently reported PTE was “online sexual violence/ abuse” (*n* = 11, 6.1%) (see Tables 2 and 3 for more detailed information). The caregivers reported a mean of 3.64 (*SD* = 2.19, range 0–11) PTEs.

**Table 2:** Prevalence rates of reported traumatic events, self- and caregiver report

Traumatic event	Self-report (*N* = 179)	Caregiver-report (*n* = 163)	Correlation between self- and caregiver-report
	*n (%)*	*n (%)*	
Natural disaster	16 (8.9)	17 (10.4)	Φ =0 .864, *p* <0 .001
Accident/injury	59 (33.0)	42 (25.8)	Φ = 0.530, *p* <0 .001
Experiencing family violence	56 (31.3)	38 (23.3)	Φ =0 .588, *p* <0 .001
Experiencing community violence	51 (28.5)	33 (20.2)	Φ =0 .679, *p* <0 .001
Attacked with weapon	17 (9.5)	12 (7.4)	Φ =0 .723, *p* <0 .001
Witnessing family violence	68 (38.0)	61 (37.4)	Φ = 0.792, *p* < 0.001
Witnessing community violence	57 (31.8)	25 (15.3)	Φ =0 .518, *p* < 0.001
Sexual violence/abuse	24 (13.4)	12 (7.4)	Φ =0 .800, *p* <0 .001
Online sexual violence/abuse	11 (6.1)	5 (3.1)	Φ =0 .618, *p* < 0.001
Bullying	71 (39.7)	58 (35.6)	Φ = 0.522, *p* < 0.001
Online bullying	22 (12.3)	11 (6.7)	Φ = 0.436, *p* <0 .001
Sudden loss	48 (26.8)	41 (25.2)	Φ =0 .812, *p* < 0.001
Scary medical procedure	46 (25.7)	40 (24.5)	Φ = 0.622, *p* < 0.001
War	123 (68.7)	119 (73.0)	Φ = 0.729, *p* < 0.001
Other not specified event	78 (49.2)	80 (49.1)	Φ =0 .681, *p* < 0.001

This data comprises the sample of children and adolescents age 7 years and older

**Table 3:** Prevalence rates of reported traumatic events in preschool children (aged 3–6), caregiver-report

Traumatic event	Total preschool sample (*n* = *21*)
	*n *(%)
Natural disaster	7 (33.3)
Accident/injury	4 (19.1)
Experiencing robbery	3 (14.3)
Experiencing family violence	4 (19.1)
Experiencing community violence	2 (9.5)
Witnessing family violence	8 (38.1)
Witnessing community violence	7 (33.3)
Being touched on private parts	1 (4.8)
Sexual violence/abuse	1 (4.8)
Sudden loss	8 (38.1)
Violent attack	3 (14.3)
Witnessing violent attack	9 (42.9)
Scary medical procedure	7 (33.3)
War	15 (71.4)
Other not specified event	13 (61.9)

For preschool children, the caregivers reported an average of 4.38 (*SD* = 2.80, range 1–10) PTEs. The most often reported PTEs were “being in a war situation” (*n* = 15, 71.4%), “witnessing a violent attack” (*n* = 9, 42.9%), “witnessing family violence” (*n* = 8, 38.1%*)* and “sudden loss” (*n* = 8, 38.1%). The least frequently reported PTEs were “Being touched on private parts” (*n* = 1, 4.8%) and “sexual violence/abuse” (*n* = 1, 4.8%).

### Posttraumatic stress disorder and complex posttraumatic stress disorder

The mean PTSS score for participants aged 7 years or older was 36.30 (*SD* = 10.40) using the DSM-5 criteria and 10.48 (*SD* = 3.96) using the ICD-11 criteria. The CPTSD score was 19.72 (*SD* = 7.06). There were no statistically significant differences in the number of reported PTEs, the PTSD and CPTSD scores between participants who were currently located in Ukraine and those who were currently in another country (see Additional file 1: Table S1). Using the caregiver report, the mean PTSS score for participants aged 7 years or older was 35.63 (*SD* = 12.06) using the DSM-5 criteria and 9.64 (*SD* = 4.59) using the ICD-11 criteria. The CPTSD score using the caregiver report was 19.03 (*SD* = 7.85) (see Table 4). For more information on differences on trauma history and PTSD scores between the genders, please see Additional file 1: Table S2.

**Table 4:** Traumatic events and PTSD symptom severity

CATS-2 self-report	Age 7 + (*n* = 179﻿) *M* (*SD*) Range	Age 3–6 (*n* = 21) *M* (*SD*) Range
Number of traumatic events	4.23 (2.54) 0–12	–
CATS-2 DSM-5 PTSD total score	36.30 (10.40) 12–58	–
CATS-2 ICD-11 PTSD total score	10.48 (3.96) 1–18	–
CATS-2 ICD-11 CPTSD total score	19.72 (7.06) 5–38	–
CATS-2 caregiver-report		
Number of traumatic events	3.64 (2.19) 0–11	4.38 (2.81) 1–10
CATS-2 DSM-5 PTSD total score	35.63 (12.06) 0–60	30.00 (5.81) 12- 40
CATS-2 ICD-11 PTSD total score	9.64 (4.59) 0–18	–
CATS-2 ICD-11 CPTSD total score	19.03 (7.85) 0–36	–

*Note. n* = 145 for the caregiver-report of the participants aged 7 or older

Table 5 shows the means of the DSM-5 and ICD-11 symptom clusters and how many participants met the respective criteria. We found that *n* = 123 (68.7%) participants fulfilled all clinical criteria for a PTSD diagnosis according to DSM-5. According to ICD-11, *n* = 56 (31.3%) fulfill the criteria for a PTSD diagnosis, *n* = 38 (21.2%) fulfilled the criteria for a CPTSD diagnosis and *n* = 85 (47.5%) did not fulfill the criteria neither for PTSD nor for CPTSD.

**Table 5:** Means, standard deviations and probable DSM-5 PTSD, ICD-11 PTSD and ICD-11 CPTSD diagnoses

		Self-report (participant age 7 or older)		Caregiver-report (participant age 7 or older)	
	Possible range	*M* (*SD*)	Criteria Met (*n*, %)	*M* (*SD*)	Criteria Met (*n*, %)
DSM-5 PTSD criteria					
Re-experiencing	0–15	9.73 (3.55)	173 (96.6)	8.96 (3.99)	149 (83.2)
Avoidance	0–6	3.70 (1.77)	141 (78.8)	3.45 (2.03)	112 (62.6)
Alterations mood /Cognitions	0–21	12.41 (4.43)	168 (93.9)	12.87 (4.59)	151 (84.4)
Hyperarousal	0–15	10.46 (3.41)	159 (88.8)	10.34 (3.94)	146 (81.6)
Functional impairment	0–5	3.58 (1.38)	178 (99.4)	3.69 (1.46)	157 (87.7)
Probable DSM-5 PTSD diagnosis			123 (68.7)		100 (55.9)
ICD-11 PTSD criteria					
Re-experiencing	0–6	3.25 (1.79)	135 (75.4)	2.81 (1.93)	109 (60.9)
Avoidance	0–6	3.70 (1.77)	141 (78.8)	3.45 (2.03)	112 (62.6)
Hyperarousal	0–6	3.53 (1.71)	141 (78.8)	3.38 (1.89)	119 (66.5)
Functional Impairment	0–5	3.58 (1.38)	178 (99.4)	3.69 (1.46)	157 (87.7)
Probable ICD-11 PTSD diagnosis			56 (31.3)		44 (24.6)
ICD-11 CPTSD criteria					
Affect dysregulation	0–6	3.65 (1.54)	150 (83.8)	4.10 (1.54)	144 (80.4)
Negative self-concept	0–6	2.41 (2.02)	75 (41.9)	2.15 (2.12)	52 (29.1)
Interpersonal disturbances	0–6	3.17 (1.95)	124 (69.3)	3.14 (1.91)	110 (61.5)
Probable ICD-11 CPTSD diagnosis			38 (21.2)		27 (15.1)
				Caregiver report (participant age 3–6)	
DSM-5 criteria					
Re-experiencing	0–15			9.81 (2.96)	20 (95.2)
Avoidance negative mood/Cognitions	0–18			10.14 (2.85)	21 (100.0)
Hyperarousal	0–15			8.10 (2.02)	21 (100.0)
Functional impairment	0–5			3.10 (1.14)	21 (100.0)
Probable DSM-5 PTSD diagnosis					20 (95.2)

Comparably, the results from the caregiver-report show that *n* = 100 (55.9%) participants fulfilled all clinical criteria for a PTSD diagnosis according to DSM-5. According to ICD-11, *n* = 44 (24.6%) fulfilled the criteria for a PTSD diagnosis, *n* = 27 (15.1%) fulfilled the criteria for a CPTSD diagnosis and *n* = 92 (60.3%) did not fulfilled the criteria neither for PTSD nor for CPTSD.

For preschool children, the mean PTSS score was 30.00 (*SD* = 5.81). In total, *n* = 20 (95.24%) met the preschool criteria for PTSD according to DSM-5.

### Comparison of self-and caregiver report

Regarding the correlation between self- and caregiver-report, there was a strong correlation between (1) the reported number of PTEs (*r* = 0.719, *p* < 0.001), (2) the CATS-2 PTSD score according to DSM-5 (*r* = 0.716, *p* < 0.001) (3) the CATS-2 PTSD score according to ICD-11 (*r* = 0.710, *p* < 0.001) and (4) the CATS-2 CPTSD score according to ICD-11 (*r* = 0.767, *p* < 0.001). Similarly, there was a moderate to high correlation between self-report and caregiver-report for the traumatic events (see Table 2 for detailed information).

## Discussion

This is the first study investigating trauma and PTSS in children and adolescents during the war in Ukraine via self- and caregiver report. The Ukrainian children and adolescents in this study reported to have experiences on average four different PTEs, which is similar to other child and adolescent samples who were in mental health treatment in Germany, Norway and the USA [15]. The most frequently reported events were similar as well (domestic violence, bullying, accident/ injury), but the Ukrainian children most frequently reported the PTE “war”. This study adds to the recent literature that Ukrainian children not only face severe war atrocities [10], but instead these experiences are often on top of many other traumatic experiences. The high number of almost 70% of the participants reporting having experienced war situations, is alarming as the long-lasting generational effects of war trauma are well-document [16]. This war might thus have a significant negative impact on an entire generation of children and adolescents.

PTSD rates were highest when applying the DSM-5 criteria with 95% of the preschool children and almost 70% of the children age 7 and older (caregiver report: 56%) meeting all criteria. This is significantly higher compared with the earlier study by Martsenkovskyi et al. [9] in which only 14.2% of the Ukrainian children age 7 and older and 18.5% of the preschool children met PTSD criteria based on the CATS caregiver report. This difference can be explained by the difference in samples though as in this study we included treatment-seeking children and in the Martenskovskyi study the sample was randomly selected independent of whether children were in any mental health treatment. The severity of the PTSD/ CPTSD symptoms (sum scores) were on average above the clinical cut-offs and much higher compared to the German, Norwegian and US treatment-seeking sample [15], which highlights the high symptom burden of Ukrainian children seeking treatment and the urgent need for a higher availability of trauma-focused treatments in Ukraine. Given similar rates of PTEs between the international samples, the higher severity scores in the Ukrainian children could potentially be explained by them experiencing an ongoing war.

The comparisons of self- and caregiver reports on trauma history and PTSD/CPTSD severity scores indicated moderate to high consistency between the patients and their caregivers. This is surprising as Sachser et al. [15] found only weak to moderate correlations between CATS-2 self- and caregiver report in the German, Norwegian and US sample. Potential explanations for the high consistency could be that Ukrainian families might communicate especially well about the child’s trauma and related stress symptoms, or parents might have an especially good understanding of their child’s symptoms as they might currently experience similar traumatic events and symptoms in light of the war [17]. Hence, it might be especially important to include these parents/ caregivers in the child’s trauma-focused treatment as this has been shown to be beneficial for parental symptomatology [18, 19].

## Future research

Although there is a large evidence-base on the effectiveness of trauma-focused treatments that could reduce trauma-related mental health disorders in children and adolescents worldwide [20], we still know very little about effective interventions in conflict-affected areas that are not considered safe environments in which we would normally implement trauma-focused treatments. Hence, first efforts in implementing and evaluating these treatments in Ukraine, such as the large-scale implementation of the “Huggy Puppy/ Hibuki Intervention” [21] in Ukraine or the TF-CBT Ukraine project [13], could help us understand how to support these children now and in the years to come.

## Limitations and strengths

Several limitations of the study need to be addressed. (1) There might be a crucial selection bias regarding the included children and adolescents as they were exclusively selected by the therapists. (2) Compared to other studies with Ukrainian children during the war [10], this study comprises a rather small sample size. Especially the pre-school sample is very small (*n* = 21), the findings for this age group should be interpreted with caution. (3) Our results might be affected by the higher rates of girls in the sample, although we couldn’t find any differences between genders regarding their trauma history, PTSD or CPTSD scores. Moreover, the gender distribution in our sample reflects the gender distribution of PTSD in children and adolescents towards girls showing higher prevalence of PTSD after experiencing traumatic events [22]. (4) We only assessed trauma and PTSD in order to keep the assessment as short and feasible as possible during the war situation, but other highly prevalent trauma-related disorders in children and adolescents (e.g. depression or anxiety), daily stressors, the region of current location inside Ukraine and displacement status should be considered in future research. (5) It is also important to note that the CATS event checklist assesses different types of traumatic events, but not the specific frequency or severity of them. Thus, the current data may underestimate the number of traumatic events experienced. Furthermore, using only the CATS questionnaire, we have only assessed one event related to war and did not distinguish between specifically war-related stressors, such as Karatzias et al. [7]. (6) The Ukrainian CATS-2 version used in this study has not been validated yet, which might question how efficient and effective the measure can be in the target population. It is of uttermost importance to translate and culturally validate internationally used and validated measures to assess mental health in Ukrainian children to better understand their current needs. Based on their needs, the national and international community can then implement tailored interventions for this population more systematically and sustainably. (7) Originally, the CATS-2 was validated for children and adolescents between 7 and 17 years of age. However, since the CATS-2 is based on and contains the ICD-11 and DSM-5 symptoms for the diagnosis of PTSD which also reflect the criteria in adults, only using a lower language level, application in young adults makes sense in the context of face validity. Nevertheless, psychometric validation for this age group is pending. (8) The study does not provide information on the parents' and other close caregivers’ psychopathology, which is especially crucial in this population as they might also be experiencing ongoing war traumatization. The parents' reports of child PTSD symptoms might however provide an indirect insight into the parent's own reaction to the trauma. (9) Although this study included parent’s report of symptoms, many other crucial factors regarding the role of the parents were not sufficiently taken into account. Among the most important factors might be parenting and its potential protective effects during (ongoing) war. In fact, a recent mixed methods systematic review and meta-analysis sheds more light on the role parenting practices play in war-exposed children and adolescent’s adjustment [23]. The authors included altogether 38 quantitative and 10 qualitative studies and found that the nature of war-related trauma affected parenting differently depending whether they were in highly dangerous settings (e.g. more harshness or hostility) compared with when living under threat (e.g. more warmth and overprotection). Future studies need to investigate parenting of Ukrainian caregivers more specifically in order to identify protective effects which could reduce or even prevent mental health difficulties in war-exposed children and adolescents in Ukraine.

Strengths of this study are: (1) The sample can be considered very heterogeneous and naturalistic as all age groups and genders could be included and recruitment took place in different locations in and outside of Ukraine. (2) The study did not solely focus on war experiences, but instead reports general life-time traumatic experiences in children and adolescents in Ukraine. (3) We used a state-of-the-art validated measure in different languages, assessing PTSD and CPTSD in self- and caregiver report.

Our study demonstrated that the Ukrainian children and adolescents seeking for treatment have experienced multiple other traumatic events in addition to war, which should be taken into account in trauma-focused psychotherapy. These children and families will face an accumulation of risk factors at different socio‐ecological levels during the ongoing war resulting in a high probability of suffering from mental health problems, calling for adequate mental health care programs for war‐traumatized communities including both individual and family level approaches [24].

### Supplementary Information

**Additional file 1: Table S1.** Differences in trauma history, PTSD and CPTSD sum scores between patients who were still in Ukraine and patients who had already left Ukraine at the measurement time point. **Table S2.** Differences in trauma history, PTSD and CPTSD sum scores between male and female patients at the measurement time point.

## Data Availability

The datasets used and/or analysed during the current study are available from the corresponding author on reasonable request.

## Abbreviations

PTSD: Posttraumatic stress disorder
CPTSD: Complex posttraumatic stress disorder
CATS: Child and adolescent trauma screen
PTE: Potentially traumatic events
PTSS: Posttraumatic stress symptoms
