# Supplementary material for: Traumatic events and posttraumatic stress symptoms in a treatment-seeking sample of Ukrainian children during the war
Source: Child Adolesc Psychiatry Ment Health. 2024 Feb 9;18:25. doi: 10.1186/s13034-024-00715-1 (PMC10858633; doi:10.1186/s13034-024-00715-1)
Supplement: Supplementary file 1 — Additional file 1: Table S1. Differences in trauma history, PTSD and CPTSD sum scores between patients who were still in Ukraine and patients who had already left Ukraine at the measurement time point. Table S2. Differences in trauma history, PTSD and CPTSD sum scores between male and female patients at the measurement time point. [file 13034_2024_715_MOESM1_ESM.docx]

**Additional 1**

**Table A**

*Differences in trauma history, PTSD and CPTSD sum scores between patients who were still in Ukraine and patients who had already left Ukraine at the measurement time point.*

|  | Inside Ukraine  (*n = 110)* | | Outside Ukraine  (*n* = 57) | | *t*(165) | *p* | Cohen’s *d* |
| --- | --- | --- | --- | --- | --- | --- | --- |
|  | *M* | *SD* | *M* | *SD* |  |  |  |
| Number of traumatic events | 4.15 | 2.76 | 4.56 | 2.23 | -0.985 | .326 | -.161 |
| DSM-5 PTSD Sum Score | 36.21 | 10.43 | 38.05 | 10.17 | -1.092 | .276 | -.178 |
| ICD-11 PTSD Sum Score | 10.57 | 3.87 | 10.81 | 4.14 | -0.362 | .718 | -.059 |
| ICD-11 CPTSD Sum Score | 19.93 | 7.01 | 20.26 | 7.04 | -0.293 | .770 | -.048 |

*Note*. This is CATS-2 self-report data.

**Table B**

*Differences in trauma history, PTSD and CPTSD sum scores between male and female patients at the measurement time point.*

|  | **Age 7+ (*n* = 179)** | | | | | | | **Age 3- 6 (*n* =21)** | | | | | | |
| --- | --- | --- | --- | --- | --- | --- | --- | --- | --- | --- | --- | --- | --- | --- |
|  | Male  (*n* = 69) | | Female  (*n* = 111) | | *t*(178) | *p* | Cohen’s *d* | Male  (*n* = 7) | | Female  (*n* = 14) | | *t*(19) | *p* | Cohen’s *d* |
|  | *M* | *SD* | *M* | *SD* |  |  |  | *M* | *SD* | *M* | *SD* |  |  |  |
| Number of traumatic events | 4.23 | 2.57 | 4.23 | 2.53 | .017 | .986 | .003 | 4.71 | 2.87 | 4.21 | 2.86 | .377 | .710 | .175 |
| DSM-5 PTSD Sum Score | 35.84 | 11.88 | 36.47 | 9.45 | -.372 | .711 | -.060 | 28.71 | 8.20 | 30.64 | 4.43 | -.708 | .244 | -.328 |
| ICD-11 PTSD Sum Score | 10.39 | 4.80 | 10.52 | 3.34 | -.199 | .842 | -.033 |  |  |  |  |  |  |  |
| ICD-11 CPTSD Sum Score | 19.28 | 7.84 | 19.95 | 6.52 | -.602 | .548 | -.096 |  |  |  |  |  |  |  |
